# Cultivating resilience in wheat: mitigating arsenic toxicity with seaweed extract and *Azospirillum brasilense*

**DOI:** 10.3389/fmicb.2024.1441719

**Published:** 2024-08-20

**Authors:** Muhammad Saqlain Zaheer, Nazish Aijaz, Akhtar Hameed, Noman Ali Buttar, Shamsur Rehman, Muhammad Waheed Riaz, Ajaz Ahmad, Muhammad Aamir Manzoor, Muhammad Asaduzzaman

**Affiliations:** ^1^Department of Agricultural Engineering, Khwaja Fareed University of Engineering and Information Technology, Rahim Yar Khan, Pakistan; ^2^School of Biomedical Science, Hunan University, Changsha, Hunan, China; ^3^MOA Key Laboratory of Soil Microbiology, Rhizobium Research Center, China Agricultural University, Beijing, China; ^4^Institute of Plant Protection, MNS University of Agriculture, Multan, Pakistan; ^5^Fundación CEAM, c/ Charles R. Darwin 14, Parque Tecnológico, Paterna, Valencia, Spain; ^6^National Key Laboratory of Wheat Improvement, Peking University Institute of Advanced Agricultural Sciences, Weifang, China; ^7^State Key Laboratory of Wheat Breeding, Group of Wheat Quality and Molecular Breeding, College of Agronomy, Shandong Agricultural University, Tai'an, Shandong, China; ^8^Department of Pharmacy, King Saud University, Riyadh, Saudi Arabia; ^9^Department of Plant Science, School of Agriculture and Biology, Shanghai Jiao Tong University, Shanghai, China; ^10^Department of Community Medicine and Global Health, Institute of Health and Society, University of Oslo, Oslo, Norway

**Keywords:** arsenic toxicity, *Azospirillum brasilense*, seaweed extract, soil organic matter, wheat growth

## Abstract

Arsenic (As) toxicity is a serious hazard to agricultural land due to growing industrialization, which has a negative effect on wheat crop yields. To address this issue, using seaweed extract and *Azospirillum brasilense* has emerged as an effective strategy for improving yield under stress conditions. However, the combined application of *A. brasilense* and seaweed extract in wheat crops under As toxicity has not been fully explored. The effectiveness of combining *A. brasilense* and seaweed extract in reducing As toxicity in wheat production was examined in this study through a 2-year pot experiment with nine treatments. These treatments included a control with no additives and two As concentrations (50 and 70 μM). At 50 and 70 μM, As was tested alone, with seaweed extract, with *A. brasilense*, and both. Significant results were achieved in reducing As toxicity in wheat crops. Arsenic at 70 μM proved more harmful than at 50 μM. The application of *A. brasilense* and seaweed extract was more effective in improving crop growth rates, chlorophyll levels, and stomatal conductance. The combined application notably decreased As concentration in wheat plants. It was concluded that applying *A. brasilense* and seaweed extract not only improves wheat growth but can also improve soil parameters under As toxicity conditions by increasing organic matter contents, boosting nutrient availability, and increasing the production of antioxidant enzymes.

## 1 Introduction

Arsenic is a chemical element that occurs naturally and is known for being hazardous for crops. It is a metalloid widely distributed in soil, water, and the environment, occurring in various forms within the Earth's crust (Fatoki and Badmus, 2022). Although arsenic has various industrial uses, its toxicity to living things, such as people and plants, makes its presence in soil and water a serious problem (Ng et al., 2003; Du et al., 2023). Arsenic is harmful to soil because plants and microbes can easily absorb and store it. Arsenic interferes with crucial plant metabolic pathways, causing toxicity (Stazi et al., 2015). Arsenic interferes with the growth and development of plants by inhibiting essential enzymes involved in photosynthesis, respiration, and nutrient intake (Sharma et al., 2020). This disruption may cause plants to develop more slowly, produce fewer crops, and have fewer vigorous plants. Arsenic can also indirectly impact the microbial communities crucial for soil fertility and nutrient cycling (Bose et al., 2022).

The toxic effects of soil-borne arsenic are particularly harmful to wheat crops, which are staple crops in many world regions. Arsenic contamination in wheat-growing areas can have various detrimental effects on wheat yield (Zhang et al., 2009; Huang et al., 2019). Arsenic-contaminated soil can prevent wheat seeds from germinating, resulting in poor plant establishment (Tong et al., 2014). After germination, arsenic-exposed wheat plants could develop stunts due to poor photosynthesis and nutrient uptake, leading to shorter plants with fewer tillers (Luo et al., 2023). Arsenic buildup in wheat grains can affect crop productivity and quality, providing health dangers to consumers, including both people and animals. Arsenic can also prevent wheat plants from absorbing important nutrients like phosphorus and iron, resulting in nutrient imbalances and deficiencies (Saeed et al., 2021). Arsenic can change the physical and chemical properties of soil, causing soil deterioration and decreasing the agricultural output in impacted areas (Saeed et al., 2021; Koley et al., 2023). Arsenic contamination in agricultural soils significantly threatens crop safety and human health. In wheat, arsenic concentrations exceeding 0.1 mg/kg are considered potentially toxic and can lead to reduced growth and yield (Rasheed et al., 2018). The toxicity of arsenic also extends to soil microbes, where concentrations above 10 mg/kg can disrupt microbial communities and affect soil health (Zecchin et al., 2021). Addressing arsenic toxicity is crucial for maintaining sustainable agricultural practices and safeguarding food quality. Recent studies have highlighted various mitigation strategies, including the use of seaweed extracts and beneficial microorganisms such as *Azospirillum brasilense*, which have shown promise in alleviating arsenic stress in crops (Zhang et al., 2009; Vezza et al., 2020; Znad et al., 2022).

Seaweed extract, made from different marine algae, can boost wheat development. Seaweed extract can be used as a biostimulant or added as fertilizer to benefit wheat crops in a number of ways. Seaweed extract also contains a range of micronutrients, including many trace elements that can be a great source of essential nutrients for plant growth (Panda et al., 2022). Adding these minerals to wheat crops via seaweed extract promotes healthier plant growth by increasing wheat biomass and grain yield (Wedad et al., 2015). Natural plant growth regulators, including auxins, cytokinins, and gibberellins, are present in seaweed extract. The ability of these substances to protect wheat plants from environmental stresses, including drought, salt, and high temperatures, is vital. Seaweed extract can potentially promote wheat plant root growth by making more effective nutrient and water uptake available (Castlehouse et al., 2003).

*Azospirillum brasilense* is a plant growth-promoting rhizobacteria (PGPR) that positively impacts soil fertility and wheat growth. PGPR has the ability to form symbiotic relationships with many plants through the root system (Helman et al., 2012). *A. brasilense* can boost the nitrogen content of the plant and make crucial nutrients for plant growth available. *A. brasilense* plays a vital role in enhancing soil fertility (Zaheer et al., 2019a; Li et al., 2023). It can increase nitrogen availability, which directly helps to increase biomass and grain output (He et al., 2023; Yi et al., 2022). *A. brasilense* can produce different compounds, such as auxins and cytokinins, that can improve plant growth through various physiological processes. PGPR can lead to a wider and more effective root system in wheat crops by uptake of more nutrients and water (Zaheer et al., 2022; Wang et al., 2024). *A. brasilense* has demonstrated potential for reducing the harmful effects of heavy metals on plant growth and physiology (Vezza et al., 2022). Heavy metal contamination of soil can poison crops like wheat, decreasing growth and output. Certain heavy metals in the soil can be mobilized by *A. brasilense*, which reduces their availability for plant absorption (Helman et al., 2012; Vezza et al., 2020). *A. brasilense* is a useful tool for sustainable agriculture, especially in regions with heavy metal-contaminated soils. *A. brasilense* can enhance wheat growth and nitrogen availability, fostering root growth and improving overall plant health (Zaheer et al., 2019b).

Arsenic toxicity significantly threatens wheat crops, adversely affecting yield and quality. While prior research has demonstrated the individual benefits of *Azospirillum brasilense* and seaweed extract in mitigating various stressors, their combined effect on arsenic-affected wheat has not been extensively investigated. This study aims to fill this gap by exploring how the synergistic application of these two agents can alleviate arsenic-induced stress in wheat. We aim to elucidate the mechanisms through which *A. brasilense* and seaweed extract interact to improve wheat resilience under arsenic stress. By advancing our understanding of these interactions, this research seeks to contribute valuable insights that could inform more effective agricultural practices for managing arsenic contamination in wheat farming. We hypothesize that leveraging the combined effects of *A. brasilense* and seaweed extract will enhance the sustainability and productivity of wheat crops affected by arsenic.

## 2 Material and methods

### 2.1 Experimental layout and crop growth conditions

Pot experiments were conducted at the Khwaja Fareed University of Engineering and Information Technology (KFUEIT), Rahim Yar Khan, Pakistan, to understand the mitigation of arsenic toxicity with seaweed extract and *Azospirillum brasilense* in wheat crops. This is the 2nd experiment of our previous research reported by Zaheer et al. (2022), in which we observed that the arsenic toxicity can be reduced with the application of *A. brasilense* and *trans-zeatin riboside* in the wheat crop, but *trans-zeatin riboside* is a chemical. Instead of using chemicals, we want to set an organic way to control arsenic toxicity, so we use seaweed extract with *A. brasilense* to control arsenic toxicity in wheat. This was a 2-year experiment with three replications in 2021–2022 and 2022–2023. An approved wheat variety from Punjab Seed Corporation, “Galaxy-2013,” was used for the experiment. *A. brasilense* was obtained from the government college (GC) university, and Lahore and seaweed (*Sargassum denticulatum*) extract was collected from the coastal areas of Pakistan. Seaweed (*Sargassum denticulatum*) was air-dried in the shadow for 10 days, and after 10 days, it dried in the oven at 60°C for 48 h. Dried seaweed was crushed in the grinder to make powder. However, 500 ml of sterilized water was added to 500 g of the seaweed powder and stored at room temperature for use as a foliar spray (Ali et al., 2022). A 2-year experiment was conducted with a complete randomized design (CRD) having nine treatments (T_0_ = control, T_1_ = As 50 μM, T_2_= As 50 μM + seaweed extract, T_3_ = As 50 μM + *A. brasilense*, T_4_ = As 50 μM + seaweed extract + *A. brasilense*, T_5_ = As 70 μM, T_6_ = As 70 μM + seaweed extract, T_7_ = As 70 μM + *A. brasilense*, T_8_ = As 70 μM + seaweed extract + *A. brasilense*). Ten kilogram of sandy loam soil was used in each pot, and this soil was mixed with different concentrations of arsenic (Na_3_AsO_4_.12H_2_O) solution as reported by Maghsoudi et al. (2020). *A. brasilense* was inoculated with wheat seeds as reported by Fukami et al. (2016) as per treatments. A total of 10 wheat seeds were sown in each pot on 20th November of each year (2021 and 2022). Seaweed extract was applied after every 20 days from the days of sowing. Irrigation water was applied at critical growth stages, and fertilizers (120–80–60 NPK kg ha^−1^) were applied uniformly after calculation for a 1-foot sq pot as per recommendation in every treatment pot. The properties of the soil used in this experiment are given in Table 1.

**Table 1:** Properties of the soil used in the experiment.

**Parameters**	**2021–2022**	**2022–2023**
Organic matter (%)	0.78	0.80
pH	7.48	7.49
EC (μS/cm)	227	228
T.S.S. (%)	0.51	0.53
Available-P (ppm)	5.15	5.18
Available-K (ppm)	112	113
Saturation percentage	35	37
**Soil separates**		
Sand (%)	37	38
Silt (%)	38	40
Clay (%)	25	22
Textural	Sandy loam	Sandy loam

### 2.2 Measured parameters

#### 2.2.1 Growth-related parameters

All crop growth-related parameters, such as plant height, spike length, spikelets per spike, grains per spike, 1,000 grain weight, and grain yield per plant, were manually calculated using a meter rod and weight balance. Crop growth rate (CGR) was observed using the following formula, as reported by Watson (1958).


$$\begin{array}{l} CGR\;\left(g/m^{2}/day\right)\;=\;\left(W_{2}-W_{1}\right)\;/\;\left(t_{2}-t_{1}\right) \end{array}$$

where W_2_, final biomass (grams) at time t_2_ (usually measured in days); W_1_: initial biomass (grams) at time t_1_; t_2_: final time (days); t_1_: initial time (days).

Relative growth rate (RGR) and net assimilation rate (NAR) were observed with the formulas reported by Willians (1946),


$$\begin{array}{l} RGR\;\left(g\;g^{-1}day^{-1}\right)\;=\;\left(\log_{e}W_{2}-\log_{e}W_{1}\right)\;/\;\left(t_{2}-t_{1}\right), \end{array}$$

where log_e_ is the natural logarithm.


$$\begin{array}{l} NAR=\;\left\{\left(W_{2}-W_{1}\right)\;/\;\left(t_{2}-t_{1}\right)\right\}\;\times \;\left\{\log_{e}L_{2}-\;log\;L_{1}/\;\left(L_{2}-L_{1}\right)\right\}, \end{array}$$

where L_1_ and L_2_ are the leaf areas at t_1_ and t_2_, respectively.

#### 2.2.2 Physiological parameters

Stomatal conductance (SC) and chlorophyll contents (CC) were observed with the use of an infrared gas analyzer (Cl-340 Handheld Photosynthesis System, USA) and chlorophyll meter (CL-1: Hansatech Instruments Ltd., UK), respectively. Relative water contents (RWC) were observed using the Barrs and Weatherley (1962) equation.


$$\begin{array}{l} RWC\;\left(\%\right)\;=\;\left[\left(FW\;-\;DW\right)\;/\;\left(TW\;-\;DW\right)\right]\;\times \;100, \end{array}$$

where FW is the fresh weight, DW is the dry weight, and TW is the turgid weight.

To calculate the electrolyte leakage (EL), samples of the leaves were placed in the test tube and incubated with distilled water at 23°C for 24 h. After shaking the test tube, electrical conductivity (EC) was noted using an EC meter. Samples were autoclaved at 60°C for 15 min, and after that, samples were cooled at 25°C and measured the EC again. EL was observed with the procedure reported by Sullivan and Ross (1979) by following the formula.


$$\begin{array}{l} EL\;Initial\;electrical\;conductivity\;=\;Final\;electrical\;conductivity. \end{array}$$

#### 2.2.3 Arsenic concentration in leaves, grains, and root

Plant samples were collected to observe arsenic (As) concentration, and any surface contaminants were removed by carefully cleaning them. Samples of leaves, grains, and roots were oven-dried at 60–70°C for 72 h and dissolved (0.25 g) in a tri-acid mixture until a clear solution as concentration was quantified with the use of ICP-AES (IRIS/AP, TJA-USA) as reported by Maghsoudi et al. (2020).

### 2.3 Soil-related parameters

Soil samples were collected at crop harvesting to analyze available N, P, K, and organic matter. Organic matter was measured using the Walkley and Black (1934) method. Soil extraction for available P was performed using sodium bicarbonate, as per Subbaiah and Asija (1956). For available K, extraction with ammonium acetate followed the method reported by Nelson and Heidel (1952). The Kjeldahl method was used to determine available N, involving digestion and subsequent titration to measure total N content. Phosphorus content was determined using spectrophotometric techniques at a wavelength of 882 nm, while potassium content was measured using flame photometry. Standard procedures were applied for calculating available N, P, and K. Soil organic carbon (SOC) was measured following the standard procedure by Sparks et al. (1996), and dissolved organic nitrogen (DON) and carbon (DOC) were determined using the method by Smolander and Kitunen (2002).

### 2.4 Statistical analysis

Data from the three replications were statistically analyzed at a 95% probability level using computer software Statistix 8.1 software and got results for two-way ANOVA. MS Excel was also used to put data into the software and to make figures.

### 2.5 Declaration of generative AI and AI-assisted technologies in the writing process

During the preparation of this manuscript the author(s) used Chat GPT and Grammarly in order to improve the technical and English language of the paper. After using this tool/service, the author(s) reviewed and edited the content as needed and take(s) full responsibility for the content of the publication.

## 3 Results

Plant height significantly affects the biological yield of the wheat crop. The highest plant height (101.33 and 100.32 cm) was observed in T_0_ under control treatment when no arsenic (As) stress was applied, followed by T_4_ (99.34 and 98.12 cm) when As stress was at the level of 50 μM and *Azospirillum brasilense* and seaweed extract were applied in the combined form, and the lowest plant height was observed in T_5_ (87.21 and 84.23 cm) when As stress was observed at the level of 70 μM with no soil amendment. The application of seaweed extract and *A. brasilense* under As stress also significantly affected spike length. The highest spike length (12.34 and 11.34 cm) was observed in T_0_ (11.33 and 10.21 cm), followed by T_4_, and the lowest spike length was observed in T_5_ (06.23 and 05.11 cm). Spikelets per spike were also negatively affected by the As toxicity, but the combined application of seaweed extract and *A. brasilense* significantly improved the growth-related parameters of wheat crops. The highest spikelets (28.32 and 26.34) were observed in T_0_, followed by T4 (26.33 and 25.12), and the lowest results were observed in T_5_ (15.12 and 15.33) in both years (Table 2).

**Table 2:** Synergistic effect of seaweed extract and *Azospirillum brasilense* on plant height, spike length, and number of spikelets per wheat spike under arsenic soil toxicity.

**Treatments**	**Plant height (cm)**		**Spike length (cm)**		**Spikelets per spike**	
	**2021–2022**	**2022–2023**	**2021–2022**	**2022–2023**	**2021–2022**	**2022–2023**
T_0_ = control	101.33 a (±0.602)	100.32 a (±0.050)	12.34 a (±0.210)	11.34 a (±0. 0.063)	28.32 a (±0. 0.063)	26.34 a (±0.086)
T_1_ = As 50 μM	94.23 de (±0.476)	93.12 e (±0.057)	09.23 d (±0.203)	08.11 d (±0.061)	20.12 e (±0. 0.798)	20.11 e (±0.093)
T_2_ = As 50 μM + seaweed extract	95.54 d (±0.573)	94.23 d (±0.045)	09.45 d (±0.211)	08.33 d (±0.078)	22.54 d (±0. 0.657)	22.12 d (±0.078)
T_3_ = As 50 μM + *A. brasilense*	97.23 c (±0.575)	96.34 c (±0.035)	10.23 c (±0.224)	09.34 c (±0.061)	24.11 c (±0. 0.532)	23.34 c (±0.086)
T_4_ = As 50 μM + Seaweed extract + *A. brasilense*	99.34 b (±0.601)	98.12 b (±0.036)	11.33 b (±0.212)	10.21 b (±0.072)	26.33 b (±0. 0.336)	25.12 b (±0.078)
T_5_ = As 70 μM	87.21 h (±0.441)	84.23 i (±0.046)	06.23 h (±0.212)	05.11 h (±0.072)	15.12 i (±0. 0.424)	15.33 i (±0.062)
T_6_ = As 70 μM + seaweed extract	88.34 g (±0.447)	86.11 h (±0.062)	06.54 g (±0.232)	05.32 g (±0.064)	16.32 h (±0. 0.524)	17.12 h (±0.092)
T_7_ = As 70 μM + *A. brasilense*	90.43 f (±0.551)	89.34 g (±0.037)	07.23 f (±0.243)	06.34 f (±0.062)	17.34 g (±0. 0.462)	18.34 g (±0.063)
T_8_= As 70 μM + seaweed extract + *A. brasilense*	92.12 e (±0.57)	91.34 f (±0.033)	08.21 e (±0.209)	07.54 e (±0.056)	19.12 f (±0. 0.063)	19.12 f (±0.078)

Different letters indicate significant differences between treatments.

Different letters indicate significant differences between treatments. Data regarding the grains per spike were significantly affected by the application of seaweed extract and *A. brasilense* (Table 2). The highest grains per spike were observed in T_0_ (49.33 and 48.12) under control treatment when there was no As toxicity and no soil amendment, followed by T_4_ (47.32 and 46.32) when As toxicity was at the level of 50 μM having the seaweed extract and *A. brasilense* application, and the lowest grains per spike were observed in T_5_ when As toxicity was at the level of 70 μM without any soil amendment in both years. Moreover, the 1,000-grain weight was also severely affected by the seaweed extract and *A. brasilense*. The highest 1,000-grain weight (39.12 and 36.12 g) was observed in T_0_, followed by T_4_ (36.43 and 34.12 g), and the lowest 1,000-grain weight was observed in T_5_ (25.12 and 23.12 g). It was also observed that the *A. brasilense* is more effective than the seaweed extract in grain yield under As toxicity, but the combined application of both amendments is more effective. The highest grain yield per plant was observed in T_0_ (1.54 and 1.41 g), followed by T_4_ (1.43 and 1.32 g), and the lowest grain yield was observed in T_5_ (0.78 and 0.52 g) in both years (Table 3).

**Table 3:** Synergistic effect of seaweed extract and *Azospirillum brasilense* on grains per spike, 1,000 grain weight, and grain yield per wheat plant under arsenic soil toxicity.

**Treatments**	**Grains per spike**		**1,000 grain weight (g)**		**Grain yield per plant (g)**	
	**2021–2022**	**2022–2023**	**2021–2022**	**2022–2023**	**2021–2022**	**2022–2023**
T_0_ = control	49.32 a (±1.028)	48.12 a (±1.321)	39.12 a (±0.644)	36.12 a (±0.598)	1.54 a (±0.066)	1.41 a (±0.0.45)
T_1_ = As 50 μM	41.22 e (±1.035)	40.12 e (±1.243)	30.33 d (±0.634)	28.23 e (±0.543)	1.12 e (±0.054)	1.02 e (±0.047)
T_2_ = As 50 μM + Seaweed extract	43.12 d (±1.087)	42.52 d (±1.245)	32.12 c (±0.624)	30.33 d (±0.532)	1.16 d (±0.056)	1.13 d (±0.052)
T_3_ = As 50 μM + *A. brasilense*	45.45 c (±0.984)	44.12 c (±1.265)	34.11 bc (±0.578)	32.44 c (±0.562)	1.26 c (±0.057)	1.24 c (±0.051)
T_4_ = As 50 μM + Seaweed extract + *A. brasilense*	47.32 b (±1.103)	46.32 b (±1.301)	36.43 b (±0.543)	34.12 b (±0.523)	1.43 b (±0.059)	1.32 b (±0.055)
T_5_ = As 70 μM	33.12 i (±1.034)	32.12 i (±1.244)	25.12 h (±0.562)	23.12 i (±0.532)	0.78 i (±0.052)	0.52 i (±0.062)
T_6_ = As 70 μM + Seaweed extract	35.21 h (±1.045)	34.66 h (±1.307)	26.45 g (±0.582)	24.65 h (±0.533)	0.83 h (±0.053)	0.58 h (±0.054)
T_7_ = As 70 μM + *A. brasilense*	37.56 g (±1.043)	36.23 g (±1.306)	27.33 f (±0.532)	25.55 g (±0.542)	0.93 g (±0.055)	0.63 g (±0.057)
T_8_ = As 70 μM + Seaweed extract + *A. brasilense*	39.23 f (±1.034)	38.12 f (±1.256)	28.64 e (±0.544)	26.54 f (±0.544)	1.00 f (±0.052)	0.76 f (±0.058)

Different letters indicate significant differences between treatments.

Crop growth rate (CGR), relative growth rate (RGR), and net assimilation rate (NAR) were negatively affected under As toxicity, but the application of seaweed extract and *A. brasilense* significantly improved the CGR, RGR, and NAR. The highest CGR (9.87 and 9.75 g m^−2^ day^−1^), RGR (4.547 and 4.423 g g^−1^ day^−1^), and NAR (4.722 and 4.643 g m^−2^ day^−1^) were observed in T_0_ when there was no As toxicity and no soil amendment, followed by T_4_ (CGR: 8.62 and 8.56 g m^−2^day^−1^, RGR: 3.921 and 3.867 g g^−1^ day^−1^, NAR: 4.233 and 4.133 g m^−2^day^−1^) when As toxicity was at the level of 50 μM with seaweed extract and *A. brasilense*. The lowest CGR (7.13 and 7.04 g m^−2^day^−1^), RGR (2.732 and 2.572 g g^−1^day^−1^) and NAR (2.633 and 2.523 g m^−2^ day^−1^) were observed in T_5_ when As toxicity was at the level of 70 μM in both years (Table 4).

**Table 4:** Synergistic effect of seaweed extract and *Azospirillum brasilense* on crop-growth rate (CGR), relative growth rate (RGR), and net assimilation rate (NAR) of wheat at flag leaf stage under arsenic soil toxicity.

**Treatments**	**CGR (g m** ^**−2**^ **day** ^**−1**^ **)**		**RGR (g g**^**−1**^ **day**^**−1**^**)**		**NAR (g m** ^**−2**^ **day** ^**−1**^ **)**	
	**2021–2022**	**2022–2023**	**2021–2022**	**2022–2023**	**2021–2022**	**2022–2023**
T_0_ = control	9.87 a (±0.050)	9.75 a (±0.062)	4.547 a (±0.043)	4.423 a (±0.051)	4.722 a (±0.025)	4.643 a (±0.033)
T_1_ = As 50 μM	8.21 e (±0.052)	8.15 e (±0.067)	3.453 e (±0.045)	3.212 e (±0.052)	3.643 e (±0.027)	3.563 e (±0.032)
T_2_ = As 50 μM + seaweed extract	8.34 d (±0.050)	8.25 d (±0.062)	3.634 d (±0.047)	3.445 d (±0.049)	3.823 d (±0.025)	3.765 d (±0.036)
T_3_ = As 50 μM + *A. brasilense*	8.48 c (±0.056)	8.35 c (±0.068)	3.823 c (±0.045)	3.621 c (±0.051)	4.021 c (±0.025)	4.011 c (±0.038)
T_4_ = As 50 μM + seaweed extract + *A. brasilense*	8.62 b (±0.062)	8.56 b (±0.059)	3.921 b (±0.042)	3.867 b (±0.053)	4.233 b (±0.026)	4.133 b (±0.038)
T_5_ = As 70 μM	07.13 i (±0.064)	07.04 i (±0.061)	2.533 i (±0.042)	2.312 i (±0.056)	2.633 i (±0.031)	2.523 i (±0.031)
T_6_ = As 70 μM + seaweed extract	07.24 h (±0.054)	07.16 h (±0.057)	2.732 h (±0.046)	2.572 h (±0.056)	2.832 h (±0.027)	2.642 h (±0.032)
T_7_ = As 70 μM + *A. brasilense*	07.36 g (±0.052)	07.23 g (±0.059)	2.942 g (±0.045)	2.733 g (±0.048)	2.912 g (±0.028)	2.865 g (±0.034)
T_8_ = As 70 μM + seaweed extract + *A. brasilense*	07.56 f (±0.058)	07.57 f (±0.061)	3.193 f (±0.042)	2.932 f (±0.047)	3.215 f (±0.021)	3.143 f (±0.034)

Different letters indicate significant differences between treatments.

Stomatal conductance (SC) was significantly affected by the application of seaweed extract and A. brasilense under arsenic (As) toxicity. Maximum SC (323 and 321 mmol m^−2^ s^−1^) was observed under control treatment (T0) when there was no As toxicity and no application of seaweed extract and *A. brasilense*, followed by T_4_ (313 and 310 mmol m^−2^ s^−1^) when As toxicity was at the level of 50 μM having the application of seaweed extract and *A. brasilense*, and the lowest SC (231 and 310 mmol m^−2^ s^−1^) was observed in T_5_ when the As toxicity was 70 μM having no application. Relative water content (RWC) and chlorophyll content (CC) were also decreased under As toxicity and improved with the application of seaweed extract and *A. brasilense*. The highest RWC (70.32 and 69.23%) and CC (52.23 and 51.23%) were observed in T_0_, followed by T_4_ (RWC: 68.88 and 67.45%, CC: 50.56 and 49.33%), and the lowest results (RWC: 54.33 and 53.12%, CC: 40.23 and 38.12%) were observed in T_5_ in both years (Table 5).

**Table 5:** Synergistic effect of seaweed extract and *Azospirillum brasilense* on stomatal conductance (SC), relative water contents (RWC), and chlorophyll contents (CC) of wheat at flag leaf stage under arsenic soil toxicity.

**Treatments**	**SC (mmol m** ^**−2**^ **s** ^**−1**^ **)**		**RWC (%)**		**CC (%)**	
	**2021–2022**	**2022–2023**	**2021–2022**	**2022–2023**	**2021–2022**	**2022–2023**
T_0_ = control	323 a (±1.452)	321 a (±1.323)	70.32 a (±0.906)	69.23 a (±0.734)	52.23 a (±0.753)	51.23 a (±0.634)
T_1_ = As 50 μM	284 e (±1.444)	281 e (±1.342)	65.32 e (±0.912)	64.33 e (±0.745)	47.34 e (±0.764)	46.32 e (±0.732)
T_2_ = As 50 μM + seaweed extract	292 d (±1.305)	289 d (±0.1.343)	66.46 d (±0.914)	65.12 d (±0.765)	48.33 d (±0.723)	47.35 d (±0.612)
T_3_ = As 50 μM + *A. brasilense*	299 c (±1.245)	297 c (±1.367)	67.34 c (±0.908)	66.13 c (±0.788)	49.34 c (±0.712)	48.35 c (±0.633)
T_4_ = As 50 μM + seaweed extract + *A. brasilense*	313 b (±1.255)	310 b (±1.355)	68.88 b (±0.908)	67.45 b (±0.789)	50.56 b (±0.721)	49.33 b (±0.644)
T_5_ = As 70 μM	231 i (±1.254)	228 i (±1.344)	54.33 i (±0.903)	53.12 i (±0.799)	39.32 i (±0.713)	37.23 i (±0.643)
T_6_ = As 70 μM + seaweed extract	236 h (±1.232)	234 h (±1.387)	55.32 h (±0.903)	54.33 h (±0.766)	40.23 h (±0.721)	38.12 h (±0.621)
T_7_ = As 70 μM + *A. brasilense*	245 g (±1.342)	242 g (±1.377)	56.34 g (±0.911)	55.56 g (±0.734)	41.34 g (±0.745)	39.34 g (±0.632)
T_8_ = As 70 μM + seaweed extract + *A. brasilense*	249 f (±1.234)	246 f (±1.379)	57.33 f (±0.921)	56.65 f (±0.732)	42.45 f (±0.745)	40.23 f (±0.641)

Different letters indicate significant differences between treatments.

**Figure 1:**
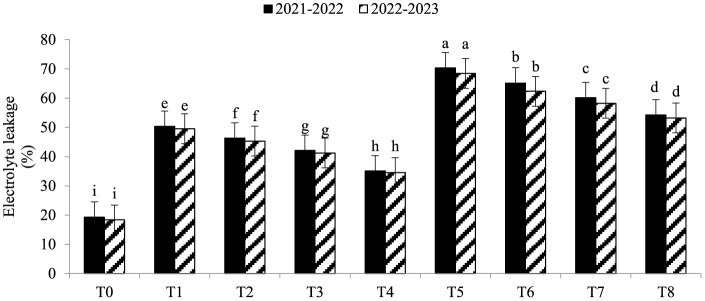
Synergistic effect of seaweed extract (SwE) and *Azospirillum brasilense* (B) on electrolyte leakage (EL) of wheat at flag leaf stage under arsenic (As) soil toxicity.

**Figure 2:**
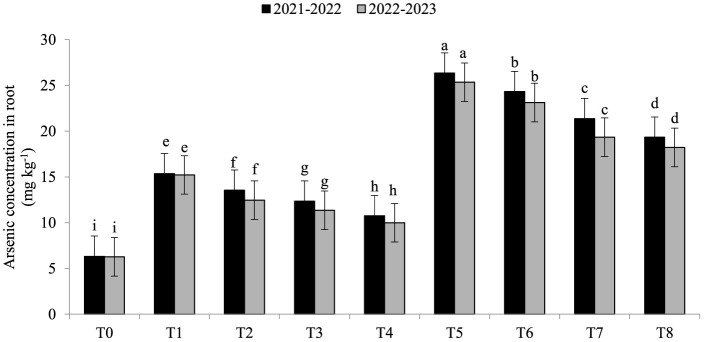
Synergistic effect of seaweed extract (SwE) and *Azospirillum brasilense* (B) on arsenic concentration in the root (mg kg^−1^) of wheat at flag leaf stage under arsenic (As) soil toxicity.

**Figure 3:**
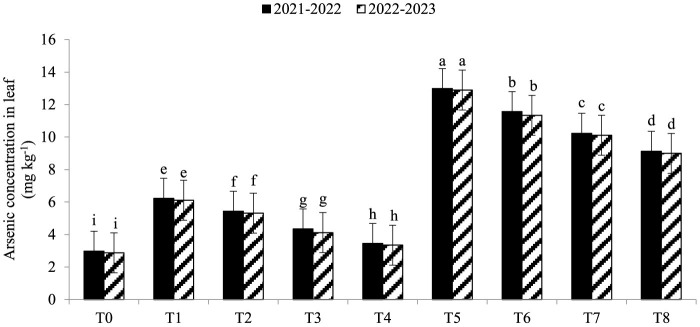
Synergistic effect of seaweed extract (SwE) and *Azospirillum brasilense* (B) on arsenic concentration in leaf (mg kg^−1^) of wheat at flag leaf stage under arsenic (As) soil toxicity.

**Figure 4:**
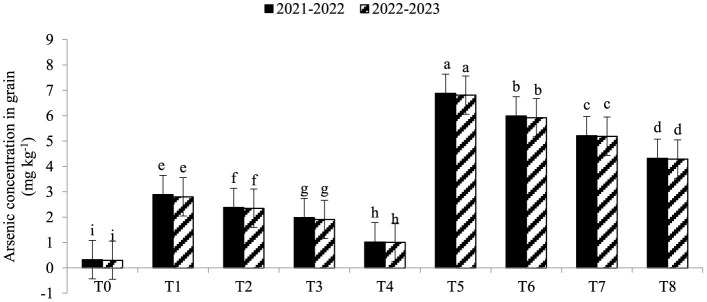
Synergistic effect of seaweed extract (SwE) and *Azospirillum brasilense* (B) on arsenic concentration in grains (mg kg^−1^) of wheat at flag leaf stage under arsenic (As) soil toxicity.

Electrolyte leakage (EL) was significantly enhanced under As toxicity and controlled with the application of seaweed extract and *A. brasilense* (Figure 1) in both years (2021–2022 and 2022–2023). Maximum EL (70.34 and 68.45%) was observed in T_5_ when As toxicity was at the level of 70 μM having no application of seaweed extract and *A. brasilense*, followed by T_6_ (65.12and 62.34%) when there was As toxicity at the level of 70 μM having the application of seaweed, and the lowest EL (19.25 and 18.34%) was observed in T0 when there was no As toxicity and no application of anything. Arsenic concentration in root, leaf, and grains also significantly increased under As toxicity, and the application of seaweed extract and *A. brasilense* was very effective in controlling their negative effect (Figures 2–4). The highest As concentration in the root (26.34 and 25.34 mg kg^−1^), leaf (12.98 and 12.89 mg kg^−1^), and grains (6.88 and 6.81 mg kg^−1^) were observed in T_5_, followed by T_6_ (As in root: 24.32 and 23.12 mg kg^−1^, As in leaf: 11.57 and 11.34 mg kg^−1^, As in grains: 5.99 and 5.92 mg kg^−1^) and the lowest (As in root: 6.32 and 6.27 mg kg^−1^, As in leaf: 2.98 and 2.87 mg kg^−1^, As in grains: 0.32 and 0.30 mg kg^−1^) was observed in T_0_ in both years.

Available N, P, and K in the soil were significantly affected by all studied treatments (Table 6). The highest available N (33.45 and 33.32 mg kg^−1^), P (16.34 and 16.25 mg kg^−1^), and K (61.34 and 58.23 mg kg^−1^) were observed under control treatment (T_0_) when there was no application of seaweed extract and *A. brasilense*, followed by T_4_ (available N: 32.33 and 31.21 mg kg^−1^, available P: 15.21 and 14.65 mg kg^−1^, available K: 57.33 and 56.43 mg kg^−1^) when there were As toxicity at the level of 50 μM having the seaweed extract and *A. brasilense*. The lowest N, P, and K were observed in T_5_ (available N: 24.56 and 23.65 mg kg^−1^, available P: 09.34 and 09.21 mg kg^−1^, and available K: 49.32 and 50.23 mg kg^−1^) when there was As toxicity at the level of 70 μM having no application of seaweed extract and *A. brasilense*. Soil organic carbon (SOC), dissolved organic carbon (DOC), and dissolved organic nitrogen (DON) are also negatively affected under As toxicity. The combined application of seaweed extract and *A. brasilense* is very effective in enhancing SOC, DOC, and DON under As toxicity (Table 7). The highest SOC (1.64 and 1.62 g kg^−1^), DOC (152.32 and 150.34 μg g^−1^), and DON (23.87 and 21.23 μg g^−1^) were observed in T_0_, followed by T_4_ (SOC: 1.45 and 1.42 g kg^−1^, DOC: 147.54 and 144.56 μg g^−1^, DON: 20.34 and 17.33 μg g^−1^), and the lowest results (SOC: 0.72 and 0.71 g kg^−1^, DOC: 123.54 and 121.23 μg g^−1^, DON: 13.45 and 12.34 μg g^−1^) were observed in T_5_ in both years.

**Table 6:** Synergistic effect of seaweed extract and *Azospirillum brasilense* on available soil nitrogen (N), phosphorus (P), and potassium (K) on wheat-grown soil under arsenic soil toxicity.

**Treatments**	**Available N (mg kg** ^**−1**^ **)**		**Available P (mg kg** ^**−1**^ **)**		**Available K (mg kg** ^**−1**^ **)**	
	**2021–2022**	**2022–2023**	**2021–2022**	**2022–2023**	**2021–2022**	**2022–2023**
T_0_ = control	33.45 a (±1.046)	33.32 a (±1.057)	16.34 a (±0.128)	16.25 a (±0.109)	61.34 a (±0.623)	58.23 a (±0.512)
T_1_ = As 50 μM	29.34 e (±1.032)	28.23 e (±1.052)	12.33 e (±0.134)	11.21 e (±0.108)	54.23 e (±0.670)	53.12 e (±0.532)
T_2_ = As 50 μM + seaweed extract	30.12 d (±1.034)	29.23 d (±1.058)	13.23 d (±0.124)	12.23 d (±0.111)	55.13 d (±0.613)	54.23 d (±0.609)
T_3_ = As 50 μM + *A. brasilense*	31.24 c (±1.053)	30.33 c (±1.053)	14.54 c (±0.128)	13.43 c (±0.112)	56.32 c (±0.622)	55.34 c (±0.576)
T_4_ = As 50 μM + seaweed extract + *A. brasilense*	32.33 b (±1.046)	31.21 b (±1.046)	15.21 b (±0.126)	14.65 b (±0.106)	57.33 b (±0.632)	56.43 b (±0.512)
T_5_ = As 70 μM	24.56 i (±1.035)	23.65 i (±1.048)	09.34 i (±0.128)	09.21 i (±0.109)	49.32 i (±0.666)	50.23 i (±0.513)
T_6_ = As 70 μM + seaweed extract	25.43 h (±1.048)	24.12 h (±1.049)	09.43 h (±0.131)	09.45 h (±0.108)	50.33 h (±0.612)	51.21 h (±0.532)
T_7_ = As 70 μM + *A. brasilense*	26.33 g (±1.049)	25.34 g (±1.047)	10.11 g (±0.134)	10.13 g (±0.108)	51.34 g (±0.631)	50.12 g (±0.532)
T_8_ = As 70 μM + seaweed extract + *A. brasilense*	27.54 f (±1.046)	26.23 f (±1.043)	11.34 f (±0.126)	10.98 f (±0.102)	52.33 f (±0.634)	51.67 f (±0.543)

Different letters indicate significant differences between treatments.

**Table 7:** Synergistic effect of seaweed extract and *Azospirillum brasilense* on soil organic carbon (SOC), dissolved organic carbon (DOC), and dissolved organic nitrogen (DON) of wheat-grown soil under arsenic soil toxicity.

**Treatments**	**SOC (g kg** ^**−1**^ **)**		**DOC (**μ**g g**^**−1**^**)**		**DON (**μ**g g**^**−1**^**)**	
	**2021–2022**	**2022–2023**	**2021–2022**	**2022–2023**	**2021–2022**	**2022–2023**
T_0_ = control	1.64 a (±0.026)	1.62 a (±0.031)	152.32 a (±1.023)	150.34 a (±1.032)	23.87 a (±0.624)	21.23 a (±0.623)
T_1_ = As 50 μM	1.20 e (±0.028)	1.19 e (±0.032)	141.23 e (±1.012)	139.23 e (±1.035)	17.32 e (±0.612)	15.34 e (±0.655)
T_2_ = As 50 μM + seaweed extract	1.29 d (±0.028)	1.28 d (±0.035)	143.45 d (±1.014)	140.21 d (±1.032)	18.34 d (±0.634)	16.34 d (±0.623)
T_3_ = As 50 μM + *A. brasilense*	1.36 c (±0.029)	1.33 c (±0.036)	145.33 c (±1.025)	142.34 c (±1.035)	19.54 c (±0.623)	16.99 c (±0.633)
T_4_ = As 50 μM + seaweed extract + *A. brasilense*	1.45 b (±0.032)	1.42 b (±0.034)	147.54 b (±1.026)	144.56 b (±1.037)	20.34 b (±0.632)	17.33 b (±0.643)
T_5_ = As 70 μM	0.72 i (±0.034)	0.71 i (±0.032)	123.54 i (±1.029)	121.23 i (±1.031)	13.45 i (±0.632)	12.34 i (±0.632)
T_6_ = As 70 μM + seaweed extract	0.81 h (±0.032)	0.79 h (±0.036)	125.23 h (±1.028)	123.45 h (±1.032)	14.34 h (±0.634)	13.45 h (±0.612)
T_7_ = As 70 μM + *A. brasilense*	0.89 g (±0.043)	0.87 g (±0.038)	127.33 g (±1.023)	124.56 g (±1.034)	15.34 g (±0.632)	14.56 g (±0.623)
T_8_ = As 70 μM + seaweed extract + *A. brasilense*	0.97 f (±0.036)	0.95 f (±0.038)	129.45 f (±1.026)	126.34 f (±1.036)	16.34 f (±0.638)	15.66 f (±0.621)

Different letters indicate significant differences between treatments.

## 4 Discussion

Seaweed extract and *Azospirillum brasilense* can have noticeable effects on plant height, spike length, and spikelets per spike, especially under arsenic (As) toxicity. These procedures present in seaweed can reduce the damaging effects of arsenic poisoning on wheat growth. Auxins, cytokinins, and gibberellins are just a few of the phytohormones found in seaweed extract that have been shown to have growth-promoting properties (Blunden et al., 1968). Seaweed extract may counteract the growth-inhibiting effects of arsenic by promoting cell elongation and general plant growth when given to wheat plants exposed to arsenic-contaminated soil (Castlehouse et al., 2003). Increased plant height could be the end outcome, resulting in wheat plants that are stronger and healthier overall. *A. brasilense* can improve wheat development by fixing atmospheric nitrogen and making more nutrients available for plants (Zaheer et al., 2019a). The presence of *A. brasilense* in arsenic-toxic soils enhances the availability of more nutrients, whereas nutrient uptake may be hindered due to As toxicity (Zaheer et al., 2022). *A. brasilense* can increase spike length and spikelet development by assuring an adequate supply of crucial nutrients and enhancing growth hormone production (Zaheer et al., 2019b). Wheat plants under arsenic stress may benefit from the synergistic application of seaweed extract and *A. brasilense*. This dual treatment, as opposed to individual treatments, may result in a more notable improvement in plant height, spike length, and spikelets per spike by improving both nutritional availability and growth-promoting chemicals (Zaheer et al., 2019b, 2022).

The production of flowers and grains may be encouraged by seaweed extract, which is a rich source of plant growth regulators, potentially boosting the number of grains per spike (Carvalho et al., 2014). This is particularly crucial in soils with arsenic contamination, where plants' development ability may be compromised (Castlehouse et al., 2003). *A. brasilense* can supplement the benefits of seaweed extract by ensuring that the plant has access to the critical nutrients required for grain growth through its capacity to improve nutrient uptake and stimulate root development (Carvalho et al., 2014; Zaheer et al., 2019a). Applying seaweed extract and *A. brasilense* together increased the number of grains per spike. Micronutrients and trace components found in seaweed extract help plants better digest nutrients, which increases grain weight (Margal et al., 2023). The ability of *A. brasilense* to fix nitrogen and solubilize nutrients can also improve grain weight (Zaheer et al., 2019a). These treatments can address nutritional deficits, resulting in heavier grains in arsenic-toxic soils. Grain yield can be significantly increased using seaweed extract and *A. brasilense*. *A. brasilense* enables effective nutrient utilization and stress tolerance (Zaheer et al., 2019b), while seaweed extract encourages plant growth and development (Begum et al., 2018). Because of this, wheat plants are more likely to have more well-developed spikes with more grains, increasing the amount of grain produced per plant.

The crop growth rate (CGR) measures how quickly a crop builds its biomass over time. Numerous growth-promoting substances, including phytohormones like auxins, cytokinins, and gibberellins, are present in seaweed extract (Blunden et al., 1968). These substances can promote cell lengthening and division, increasing the biomass produced by wheat plants. A crucial nutrient for plant growth can be made more readily available by *A. brasilense*. By encouraging cell growth and nutrient supply in As-contaminated soil, using seaweed extract and *A. brasilense* together can considerably increase CGR (Castlehouse et al., 2003; Zaheer et al., 2019a). Relative Growth Rate (RGR) is an indicator for evaluating how quickly a plant's biomass increases compared to its starting biomass. Wheat plants can better withstand the negative effects of arsenic toxicity because of the antioxidants and other beneficial substances found in seaweed extract (Deolu-Ajayi et al., 2021). *A. brasilense* can boost nutrient uptake and root growth (Zaheer et al., 2019a), which helps to improve plant vigor and, as a result, increase RGR (Zhang et al., 2023a). This indicates that treated wheat plants are utilizing their resources more effectively while developing quickly. The effectiveness of a plant in turning ingested carbon dioxide into biomass is indicated by its net assimilation rate (NAR). Seaweed extract can enhance NAR by enhancing photosynthetic efficiency, expanding the supply of carbon skeletons, and providing more energy for growth (El-Din, 2015). *A. brasilense* can support NAR by enhancing nutrient uptake, particularly nitrogen, which is crucial for photosynthesis and biomass production (Zaheer et al., 2019a). Combining these treatments in arsenic-contaminated soils can result in a more effective use of resources and a higher NAR. Stomatal conductance (SC) is a key factor governing the exchange of gases, mainly carbon dioxide and water vapor. Frequently decreased stomatal conductance was observed under arsenic toxicity due to the water loss (Bali and Sidhu, 2021). Seaweed extract can enhance SC by possessing certain chemicals that increase plants' tolerance to abiotic stresses. These substances can support or improve stomatal conductance, enabling the plant to continue photosynthesizing effectively even under challenging circumstances (Deolu-Ajayi et al., 2021; Zhao et al., 2023). *A. brasilense* can increase SC by ensuring the plant has enough water necessary for stomatal opening through its influence on root development and nutrient uptake (Zaheer et al., 2022). Plants often find it difficult to maintain relative water content (RWC) in arsenic-contaminated soils due to the damaging impact of arsenic on water intake and plant transport mechanisms. Osmoprotectants and antioxidants found in seaweed extract can help plants retain RWC by reducing the cellular damage brought on by arsenic (Saad-Allah and Nessim, 2016). *A. brasilense* can also help RWC by improving the plant's capacity for water absorption and retention and fostering root growth and nutrient uptake (Zaheer et al., 2019a). Higher As toxicity can lead to a higher reduction in crop growth (Zaheer et al., 2022), and the combined application of seaweed extract and *A. brasilense* is very effective in controlling the negative effect of As toxicity on wheat growth (Deolu-Ajayi et al., 2021; Zaheer et al., 2022). An important sign of a plant's photosynthetic activity and general health is its chlorophyll concentration. As arsenic exposure interferes with photosynthetic activities, chlorophyll concentration frequently decreases (Marcelle et al., 1983). Rich in micronutrients and growth-promoting substances, seaweed extract can boost chlorophyll synthesis and shield chloroplasts from arsenic-induced oxidative damage (Sati et al., 2021). Additionally, *A. brasilense* might indirectly improve CC by increasing the availability of nutrients, especially nitrogen, which is necessary for the synthesis of chlorophyll (Wang et al., 2020; Peng et al., 2021). To maintain or improve CC in wheat plants growing in arsenic-contaminated soil, seaweed extract and *A. brasilense* are used in combination and are very effective.

Seaweed extract and *A. brasilense* can greatly impact how much electrolyte leakage (EL) is in wheat plants cultivated in As-contaminated soil. Electrolyte leakage is a gauge of how well-maintained a plant cell membrane is (Bajji et al., 2002). Cell membrane damages when exposed to As toxicity due to stress conditions that can cause the leakage of electrolytes in plant cells (Hryvusevich et al., 2022). Antioxidants found in seaweed extracts are among the many bioactive substances that can help shield cell membranes from oxidative harm caused by arsenic toxicity. These substances have the capacity to lessen the degree of membrane permeability and the ensuing electrolyte leakage (Deuticke et al., 1987). Osmoprotectants are frequently present in seaweed extracts and might improve a plant's capacity to preserve membrane integrity under adverse conditions (Deolu-Ajayi et al., 2021). *A. brasilense* can decrease electrolyte loss by improving plant health by producing more growth hormones, especially cytokinin, which is helpful in cell division (Zaheer et al., 2022). *A. brasilense* improves nutrient uptake and root growth, both of which help the plant respond to arsenic stress more skillfully (Zaheer et al., 2019b). Wheat plants exposed to arsenic poisoning may experience less electrolyte leakage due to the presence of *A. brasilense* in the soil.

Arsenic concentrations in various areas of wheat plants growing in soil contaminated with arsenic toxicity can be greatly influenced by seaweed extract and *A. brasilense*. Arsenic ions in the soil can be bound by seaweed extract substances, making them less available for plant roots to absorb. Seaweed extracts have the ability to promote root growth, which reduces arsenic uptake by allowing the roots to penetrate less contaminated soil areas (Khan et al., 2009). *A. brasilense* lowers the concentration of arsenic in the roots by increasing the nutrient uptake and dilution of As concentration in the soil (Zaheer et al., 2022). Seaweed extract can reduce the stress caused by arsenic in plants through its antioxidants and stress-response components, resulting in healthier leaves with less arsenic buildup (Castlehouse et al., 2003; Khan et al., 2009). *A. brasilense* helps lower leaf arsenic concentration as it makes a nutrient barrier to uptake less As from the soil, which can assist in reducing arsenic levels (Gomes et al., 2012). Food safety requires lowering the content of arsenic in grains. Seaweed extract promotes grain development and guards against stress brought on by arsenic, resulting in healthier grains that accumulate less arsenic (Castlehouse et al., 2003; Khan et al., 2009). The ability of *A. brasilense* to withstand stress and absorb nutrients may also reduce the amount of arsenic in grains (Zaheer et al., 2019b, 2022). The mechanism by which seaweed extract and *A. brasilense* mitigate arsenic toxicity involves multiple pathways. Seaweed extract is rich in bioactive compounds, such as polysaccharides and antioxidants (Castlehouse et al., 2003; Khan et al., 2009), which enhance plant tolerance to stress by scavenging reactive oxygen species and improving nutrient uptake. *A. brasilense* enhances root growth and nutrient assimilation, facilitating better arsenic detoxification and reducing uptake (Zaheer et al., 2022). Both treatments improve plant resilience and growth under arsenic stress, as observed in our results.

The solubility and mineralization of nutrients in the soil are improved by the chemicals found in seaweed extract, which increases the availability of nitrogen, phosphorus, and potassium (Zaheer et al., 2022; Zhang et al., 2023b). Applying seaweed extract can offer a conveniently available source of these crucial nutrients to assist wheat plant growth and development in arsenic-contaminated soils (Castlehouse et al., 2003; Cheng et al., 2024). *A. brasilense* contributes to increased nutrient availability in the soil through its function in root growth and nutrient uptake. By fixing atmospheric nitrogen and making it available to plants, this advantageous bacterium can help improve nitrogen availability (Zaheer et al., 2022). When seaweed extract and *A. brasilense* are used together, wheat plants receive access to the critical NPK nutrients.

Organic material found in seaweed extract can raise soil levels of organic carbon (SOC) and dissolved organic carbon (DOC). This organic material acts as a substrate for microbial activity once absorbed into the soil, encouraging the growth of advantageous soil microbes (Witzgall et al., 2021; Qiu et al., 2023). These microbes contribute to the buildup of SOC and DOC by decomposing organic materials. *A. brasilense* can encourage the breakdown of organic compounds, boosting SOC and DOC levels even further (Cook et al., 2022). Applying these treatments can aid in restoring and maintaining SOC and DOC, which are essential for soil structure, water retention, and nutrient cycling, in arsenic-contaminated soils where microbial activity may be impeded (Pal, 2020; Wu et al., 2024). The decomposition of organic matter and microbial activity in the soil is directly related to the availability of dissolved organic nitrogen (DON). By encouraging microbial development and improving nutrient cycling, seaweed extract, and *A. brasilense* can boost the release of DON into the soil solution. To provide a conveniently accessible nitrogen supply for plant absorption, DON must exist.

## 5 Conclusion

Arsenic toxicity significantly impairs wheat crop growth, exacerbating adverse effects as soil arsenic levels rise. This study highlights the beneficial impact of *Azospirillum brasilense* and seaweed extract in alleviating arsenic toxicity and promoting healthier, more resilient wheat plants. Both *A. brasilense* and seaweed extract individually enhance plant health by improving nutrient uptake, stimulating growth rates, and increasing antioxidant enzyme production, which helps plants cope with stress. The combined application of *A. brasilense* and seaweed extract proves particularly effective in mitigating arsenic toxicity. This synergy results in a more pronounced reduction in arsenic uptake by wheat plants, leading to better overall plant health and productivity.

The combination also enhances soil quality by increasing organic matter content and nutrient availability, fostering a more fertile and sustainable growing environment. The integrated use of *A. brasilense* and seaweed extract offers a robust, organic solution to managing arsenic toxicity in wheat crops. This approach not only supports sustainable agriculture by reducing reliance on chemical treatments but also provides a resilient strategy for maintaining wheat yield and quality in arsenic-contaminated soils. The findings of this study suggest that adopting such practices can significantly benefit farmers and agricultural communities, ensuring the long-term viability and productivity of wheat farming in regions affected by arsenic contamination.

## Data Availability

The raw data supporting the conclusions of this article will be made available by the authors, without undue reservation.
